# Swimming with Predators and Pesticides: How Environmental Stressors Affect the Thermal Physiology of Tadpoles

**DOI:** 10.1371/journal.pone.0098265

**Published:** 2014-05-28

**Authors:** Marco Katzenberger, John Hammond, Helder Duarte, Miguel Tejedo, Cecilia Calabuig, Rick A. Relyea

**Affiliations:** 1 Department of Evolutionary Ecology, Doñana Biological Station - Spanish Council for Scientific Research, Sevilla, Spain; 2 Department of Biology, University of New Mexico, Albuquerque, New Mexico, United States of America; 3 Department of Animal Sciences, Federal Rural University of the Semiarid Region, Mossoró, Rio Grande do Norte, Brazil; 4 Department of Biological Sciences, University of Pittsburgh, Pittsburgh, Pennsylvania, United States of America; Clemson University, United States of America

## Abstract

To forecast biological responses to changing environments, we need to understand how a species's physiology varies through space and time and assess how changes in physiological function due to environmental changes may interact with phenotypic changes caused by other types of environmental variation. Amphibian larvae are well known for expressing environmentally induced phenotypes, but relatively little is known about how these responses might interact with changing temperatures and their thermal physiology. To address this question, we studied the thermal physiology of grey treefrog tadpoles (*Hyla versicolor*) by determining whether exposures to predator cues and an herbicide (Roundup) can alter their critical maximum temperature (CT_max_) and their swimming speed across a range of temperatures, which provides estimates of optimal temperature (T_opt_) for swimming speed and the shape of the thermal performance curve (TPC). We discovered that predator cues induced a 0.4°C higher CT_max_ value, whereas the herbicide had no effect. Tadpoles exposed to predator cues or the herbicide swam faster than control tadpoles and the increase in burst speed was higher near T_opt_. In regard to the shape of the TPC, exposure to predator cues increased T_opt_ by 1.5°C, while exposure to the herbicide marginally lowered T_opt_ by 0.4°C. Combining predator cues and the herbicide produced an intermediate T_opt_ that was 0.5°C higher than the control. To our knowledge this is the first study to demonstrate a predator altering the thermal physiology of amphibian larvae (prey) by increasing CT_max_, increasing the optimum temperature, and producing changes in the thermal performance curves. Furthermore, these plastic responses of CT_max_ and TPC to different inducing environments should be considered when forecasting biological responses to global warming.

## Introduction

Biological mechanisms underlying a response to environmental changes can be quite complex. To forecast these biological responses, we need to understand how a species' physiology varies through space and time [1], [2] and assess how changes in physiological function induced by environmental changes (e.g., increasing environmental temperatures) may interact with phenotypic changes induced by other types of environmental variation [3], [4], [5], [6].

Species can possess the ability to respond to new or altered environments with flexible phenotypes that are environmentally induced and can potentially contribute to adaptive evolution [7]. Stressful environments can induce non-adaptive plasticity, increasing the variance around the mean phenotypic response or distancing it from the favored optimum. Nevertheless, if plasticity is adaptive and promotes establishment and persistence in a new environment, by placing populations close enough to a new phenotypic optimum for directional selection to act, it can predictably enhance fitness and is most likely to facilitate adaptive evolution on ecological timescales [7].

The presence of predators in the environment can induce behavioral and morphological changes in prey that result in the prey being less susceptible to the predator (e.g., [8], [9], [10], [11]). Furthermore, pesticides can also induce behavioral and morphological changes in organisms. Sublethal exposure to pesticides early in life can make the individuals more tolerant of the pesticide later in life [12], [13] and they can induce phenotypic changes that resemble predator-induced phenotypes [14], [15], [16], [17]. In other cases, pesticides impede the induction of predator-induced morphology [18], [19], [20], [21].

In the current scenario of climate change, there has been a renewed interest in the thermal physiology of organisms and the estimation of thermal tolerance and sensitivity, using physiological traits such as the critical thermal maximum (CT_max_; e.g., the temperature at which animals become immobile [22], [23]), the optimum temperature (T_opt_) for performing some function, or the shape of the thermal performance curve (TPC), which describes how an animal's performance changes across a range of temperatures. Although some pesticides are known to affect CT_max_ and burst speed, usually in a negative way (e.g., [24]), there is limited information on how pesticides affect optimum temperature and performance over a range of temperatures (i.e. how pesticides affect TPCs), especially for amphibians. Likewise, much is known about predator-induced changes in organisms, including some interactions with pesticides [17]. Predators also influence thermoregulation and thermal preferences of prey, resulting in behavioral changes and coevolution of thermal optima between species [25]. Other than these behavioral responses that indirectly affect physiology, little is known about whether predator cues can directly affect the thermal physiology of prey.

We addressed these issues by studying the thermal physiology of grey treefrog tadpoles (*Hyla versicolor* LeConte 1825) that were exposed to predator cues and pesticides. Tadpoles are excellent model organisms for this study because they are practically isothermal with their aquatic environment [23] and their thermal physiology traits (CT_max_ and T_opt_) are not influenced by confounding processes such as dehydration. Tadpoles are also well known for expressing predator-induced changes in behavior and morphology (e.g., [9], [26], [27]. Furthermore, at least two species of tadpoles can alter their morphology when exposed to the herbicide Roundup and exhibit morphological changes that closely resemble predator-induced changes in tadpoles [17].

Given that pollutants and predators can both affect many aspects of tadpole biology, including development and metamorphosis (e.g., [28], [29]), and the interaction of pollutants with other stressors are often negative to the organism (e.g., glyphosate, [30]), we expect the impact of these stressors on the thermal physiology of tadpoles to be mainly negative. Therefore, we hypothesized that tadpoles exposed a sublethal concentration of an herbicide will have reduced tolerance to higher temperatures (CT_max_) and exhibit a lower optimal temperature (T_opt_) compared to tadpoles not exposed to the herbicide. Furthermore, because predator cues and the herbicide can induce deeper tails in tadpoles, we hypothesized that tadpoles exposed to either stressor will suffer a vertical shift upward in their TPC across a range of temperatures [31], and have increased swimming performance (e.g., [32]). However, it is also possible that the herbicide will have a negative effect on swimming performance (e.g., [33]) if induced morphological changes are countered by other phenotypic changes that impair swimming ability.

## Methods

### Inducing the tadpoles

The induction experiment was conducted at the University of Pittsburgh's Pymatuning Laboratory of Ecology in northwest Pennsylvania, USA. The experiment used a completely randomized, 2×2 factorial design comprised of the presence or absence of predator cues crossed with the presence or absence of an herbicide (nominal concentrations of 0 or 2 mg active ingredient per liter (a.e./L). Based on past studies, this herbicide concentration should remain sublethal to gray treefrog tadpoles while inducing morphology changes (e.g., [34], [35]).

The four treatment combinations were replicated four times for a total of 16 mesocosms, which consisted of 120-L wading pools, set outdoors (air temperature ranged from 9°C to 28°C), that we filled with 100 L of well water on 11 June 2011. We then added 100 g of dry leaves (*Quercus* spp.) and 5 g of rabbit chow to serve as habitat structure and an initial nutrient source, respectively. We also added an aliquot of zooplankton and phytoplankton that was a mixture from 5 local ponds. Each mesocosm was equipped with a predator cage constructed of 10×10 cm well pipe covered with window screen at each end. These cages allow the chemical cues emitted during predation to diffuse through the water while preventing the predators from killing the target tadpoles [36], [37], [38]. Mesocosms were covered with a 60% shade cloth, for the duration of the outdoor experiment.

To obtain tadpoles for the experiment, we collected >20 amplecting pairs of grey treefrogs from a nearby wetland (41° 34′ 9.55" N, 80° 27′ 22.29" W) on 18, 21 and 22 May 2011, and allowed them to lay eggs in tubs containing aged well water. Once the eggs hatched, the tadpoles were held in outdoor pools and fed rabbit pellets *ad libitum* until used in the experiment.

On 15 June 2011, which we defined as day 0 of the experiment, we added 40 tadpoles to each mesocosm from a mixture of the clutches with an initial mass (±SE) of 37.5±2.1 mg per tadpole (subsample, N = 20). On day 1, we applied the herbicide treatment. To achieve nominal concentrations of 2 mg a.e./L, we prepared 8 equal mixtures containing 372 µL of stock solution (Roundup Power Max; concentration  = 540 g a.e./L) and 250 ml of water. For the eight mesocosms assigned the herbicide treatment, we drizzled one mixture into each mesocosm. For the eight mesocosms assigned the no-herbicide treatment, we drizzled 250 mL of water into each mesocosm. Approximately 1 hr after dosing, we collected water samples from each tank to confirm the concentration of the herbicide. An independent analysis found that the concentrations in the water were 0 and 1.55 mg a.e./L (Mississippi State Chemical Laboratory, Mississippi State, MS). Observing lower actual concentrations is a common phenomenon in mesocosm experiments (reviewed in Brock et al. 2000), likely as the result of binding to surfaces in the mesocosm and degradation of the samples before the testing is conducted. Jones et al. [39] measured little herbicide breakdown for a similar time period, so we assumed there was little change in herbicide concentration during the induction experiment.

After sampling the water, we manipulated the predator environment. For mesocosms assigned the no-predator treatment, the cages remained empty. For mesocosms assigned the predator-cue treatment, we placed a single dragonfly nymph (*Anax junius*) inside the predator cage. Each dragonfly was fed ∼300 mg of grey treefrog tadpole biomass every 2 d (see [38]). Prior to each feeding, we observed no tadpoles left in the predator cage, which indicates that the dragonfly nymphs consumed the tadpoles in the cages. The feeding continued until day 10 to allow tadpole growth and induction by the herbicide and predator cues.

### Determining the critical thermal maximum of the tadpoles

On day 10, we brought sets of tadpoles into the laboratory to allow them to acclimate at a temperature of 20°C (approximately the average temperature experienced in the mesocosms), with a 12L:12D photoperiod, for 4 to 5 d before testing them for CT_max_ and T_opt_ [22], [40]. During acclimation, tadpoles were fed rabbit pellets *ad libitum* and we maintained the predator and herbicide environments to help prevent the loss of any phenotype induction [41]. All tested larvae were below Gosner stage 38 [42]. This is important because tadpoles close to metamorphic climax exhibit a significant decline in thermal tolerance [43].

We obtained upper critical thermal tolerances (CT_max_) by using a slightly modified version of Hutchison's dynamic method [23]. We exposed tadpoles to a constant heating rate of 0.05°C min^−1^ (3°C h^−1^), which simulates a natural rate of temperature increase in ponds (H. Duarte, M. Tejedo, J. Hammond, M. Katzenberger, R.A. Relyea, unpublished data from dataloggers; see also [44]) until we observed complete immobility, which signaled the endpoint of the experiment. After reaching CT_max_, we transferred tadpoles to cooler water (∼20°C) to allow recovery. After complete recovery, the tadpoles were weighed and we found that the mass of the tadpoles had increased by 13- to 15-fold since day 0. We tested 3 to 4 tadpoles from each mesocosm, for a total of 56 tadpoles from the 16 mesocosms, as seen in Table 1.

**Table 1:** Critical thermal maximum (CT_max_), sample size (N) and body mass (Mass) of *Hyla versicolor* tadpoles, in four treatments.

Treatment	N	CT_max_ (°C±SE)	Mass (mg±SE)
**Control**	13	41.78±0.1	483.7±22.9
**Predator**	13	42.14±0.1	520.4±29.3
**Roundup**	15	41.76±0.1	545.4±28.0
**Predator + Roundup**	15	42.17±0.1	489.8±34.2

Tested tadpoles are representative of the four mesocosms used for each treatment.

We performed an analysis of variance (ANOVA) that used CT_max_ as the dependent variable, predator cues and herbicide as categorical factors (including the interaction of these factors), and mesocosm nested within the interaction of predator cues and herbicide (i.e. mesocosm nested within treatment). Given that tadpole mass was not correlated with CT_max_ (see results), we did not include it as a covariate. No data transformations were required for this analysis.

### Determining the thermal performance curves for tadpole burst speed

Locomotor performance, measured as a TPC, is considered to be a proxy of maximum physiological performance and has been used to estimate optimum temperatures in amphibians [45], [46]. We obtained TPCs by measuring each tadpole's maximal burst swimming speed (i.e. burst speed) across a range of temperatures. To determine burst speed, tadpoles were placed individually in a portable thermal bath (patent license ES 2372085), which consists of an opened cross section methacrylate tube (1 m long ×6 cm wide ×3 cm deep) filled with water of a given temperature. We then gently prodded the tadpole with a thin stick to stimulate swimming. Each trial was recorded using a digital camera (30 frames/s) positioned above the tube (JVC Everio GZ-MG505). TPCs were defined using a set of six temperatures (20°, 24°, 28°, 32°, 35° and 38°C). This set includes temperatures tadpoles experienced in the mesocosms (20°-32°C) and two more (35° and 38°C) which they might be exposed to in a scenario of increasing environmental temperatures (but lower than their critical thermal maximum). Temperatures were tested in a random order and, for each temperature, tadpoles from the four treatments were tested in the same session; therefore, all treatments had the same temperature order. Prior to swimming, tadpoles were held individually in 250-ml containers at the test temperature for approximately 1 hr. A different set of tadpoles (total N = 570) was used for each temperature (Table 2) and each wading pool was represented equally in each set.

**Table 2:** Experimental temperatures, sample size (N), total tadpole length in mm (TTL±SE), and maximum swimming speeds in cm/s (mean ±SE) for gray treefrog tadpoles that were exposed to predator cues and the herbicide Roundup.

Temp.	Control			Predator			Roundup			Predator + Roundup		
	N	TTL	Speed	N	TTL	Speed	N	TTL	Speed	N	TTL	Speed
20°C	22	37.3±0.6	39.2±1.0	24	38.2±0.6	41.1±1.1	23	38.8±0.6	41.0±1.4	24	37.9±0.7	40.2±1.3
24°C	24	38.1±0.5	41.3±1.1	24	41.0±0.5	46.9±1.5	23	38.8±0.5	44.0±1.3	23	38.0±0.6	44.0±1.3
28°C	24	39.9±0.6	45.4±1.5	24	42.1±0.5	52.3±1.7	24	41.8±0.5	50.7±1.8	24	39.7±0.6	50.3±1.7
32°C	24	39.4±0.6	46.7±1.3	24	39.7±0.5	52.5±1.2	24	40.2±0.6	50.2±1.4	24	39.1±0.6	52.5±1.1
35°C	24	39.6±0.6	45.8±1.7	24	40.8±0.5	51.5±1.6	24	40.6±0.5	47.1±1.7	24	40.4±0.6	50.1±1.8
38°C	24	37.9±0.5	40.2±1.8	24	37.6±0.6	44.6±1.6	24	39.1±0.6	41.5±2.1	23	36.2±0.6	42.3±2.1

After the tadpole started to move, we used the software Measurement in Motion [47] to estimate burst speed over three frames (0.1 s) by measuring the distance the center of mass moved between frames [48], [49]. After conducting at least three bouts, we used the fastest speed measured for a given tadpole as our measure of that individual's burst speed. Since maximal swimming speed may scale with body size [45] and body size may confound the effect of speed on escape success [50], we used size-corrected burst speed (using tadpole total length) when constructing TPCs.

To describe the TPCs for burst speed, we used the Template Mode of Variation method (TMV, [51]) which employs a polynomial function to decompose variation among TPCs into three predetermined modes of variation with biological connotation: vertical shift (faster-slower), horizontal shift (hotter-colder), and specialist-generalist trade-offs ([31]; see [51] and supporting information for details on calculations). Since we tested tadpole performance at six temperatures, we assumed that the common template curve was a fourth-degree polynomial, as in previous studies (e.g., [46]). Making this assumption avoids inadequately describing TPCs, which can happen when using a lower-order polynomial [51], [52].

In addition to using the TMV method, we also calculated maximum performance (z_max_) to evaluate changes in maximum swimming speed at the optimum temperature and a more traditional measurement of performance breadth to confirm specialist-generalist trade-offs (using B_95_, which is the range of temperatures at which performance values exceed 95% of the maximum;[53]). We used B_95_ instead of the traditional B_80_ because the lower limit of B_80_ would fall below 20°C, which is outside the tested range of temperatures. All computations regarding the TPCs, except for B_95_, were made using the Matlab code by R. Izem (available online in the appendix of [51]). We also confirmed the fit of each treatment's curve and calculated standard error (SE) of each curve's parameters using nlinfit and nlparci functions, respectively, in Matlab [54].

We conducted an ANOVA that used burst speed as the dependent variable, temperature, predator cues and the herbicide (including the interaction of these factors) as categorical factors and, mesocosm nested within the interaction of predator cues and herbicide (i.e. mesocosm nested within treatment). ANOVA analysis was followed by a Tukey post-hoc test.

### Assessing the morphology of the tadpoles

After the swimming trials, we determined the mass and developmental stage of each tested tadpole. We then took lateral photos of each tadpole and digitized the images for morphometric measurements. We captured the shape of tadpoles by digitizing 10 landmarks and 15 semi-landmarks (see supporting information; see also [49], [55]) on each tadpole using tpsDig2 software [56]. We then extracted partial warps and the uniform component with tpsRelw software [57], which we used as our shape variables in a subsequent analysis. We visualized variation in landmark positions using the thin-plate spline approach (transformation grids, [58] in MorphJ [59]. As an alternative approach to quantify tadpole morphology, we also took the following linear measurements of each tadpole: total tadpole length (TTL, distance between snout and tip of tail fin), body length (BL, distance between snout and point where bottom edge of tail muscle meets body), body depth (BD, deepest point of the body), tail length (TL, distance between point where bottom edge of tail muscle meets body and tip of tail fin), muscle depth (MD, deepest point of the muscle) and tail depth (TD, maximum depth of the tail fin).

We conducted canonical correlation analysis as a dimension-reducing procedure to obtain two morphological indices (i.e. a linear combination of shape variables); one was for the linear measurements (MI_lin_) and the other was for the partial warps and uniform component (MI_geo_). We then examined these two indices for correlations with burst speed (across all treatments; see [55]). To determine if predator cues, herbicide, and their interaction influenced tadpole size (i.e. centroid) or shape (MI_lin_ or MI_geo_), we performed three ANOVAs followed by Tukey HSD post-hoc tests; mesocosms were nested within the interaction of predator cues and herbicide (i.e. mesocosm nested within treatment). Shape variables (MI_lin_ and MI_geo_) and tadpole size (centroid) were then used as continuous predictors, along with temperature, predator cues and herbicide as a categorical predictors, in two ANCOVA analysis (testing either MI_lin_ or MI_geo_ separately), to evaluate their effects on burst speed. We performed all analyses using Matlab [54], except when mentioned otherwise, and used a significance level of α = 0.05.

All experiments were approved by the University of Pittsburgh's Institutional Animal Care and Use Committee (Protocol #12050451).

## Results

### Critical thermal maxima of the tadpoles

In our analysis of CT_max_, there were no differences among mesocosms within a given treatment. We found an effect of predator cues but no effect of the herbicide or the interaction of both (Table 3). Averaged across herbicide treatments, tadpoles exposed to predators had a CT_max_ that was 0.4°C higher than tadpoles not exposed to predators (Table 1). CT_max_ was not correlated with tadpole mass (Pearson's R = −0.17, *p* = 0.22).

**Table 3:** ANOVA using CT_max_ as dependent variable, predator cues and Roundup as categorical factors (including the interaction of these factors) and, mesocosm nested within the interaction of predator cues and Roundup, for *Hyla versicolor*.

	SS	d.f.	MS	F	p
**Predator**	1.993	1	1.993	14.9	<0.001
**Roundup**	0.006	1	0.006	0.04	0.834
Predator*Roundup	0.009	1	0.009	0.06	0.801
Mesocosm (Predator*Roundup)	1.329	12	0.111	0.83	0.622
**Error**	5.350	40	0.134		

Univariate tests of significance for CT_max_. In this model, we used Sigma-restricted parameterization and Type III sum of squares.

### Thermal performance curves for tadpole burst speed

**Figure 1:**
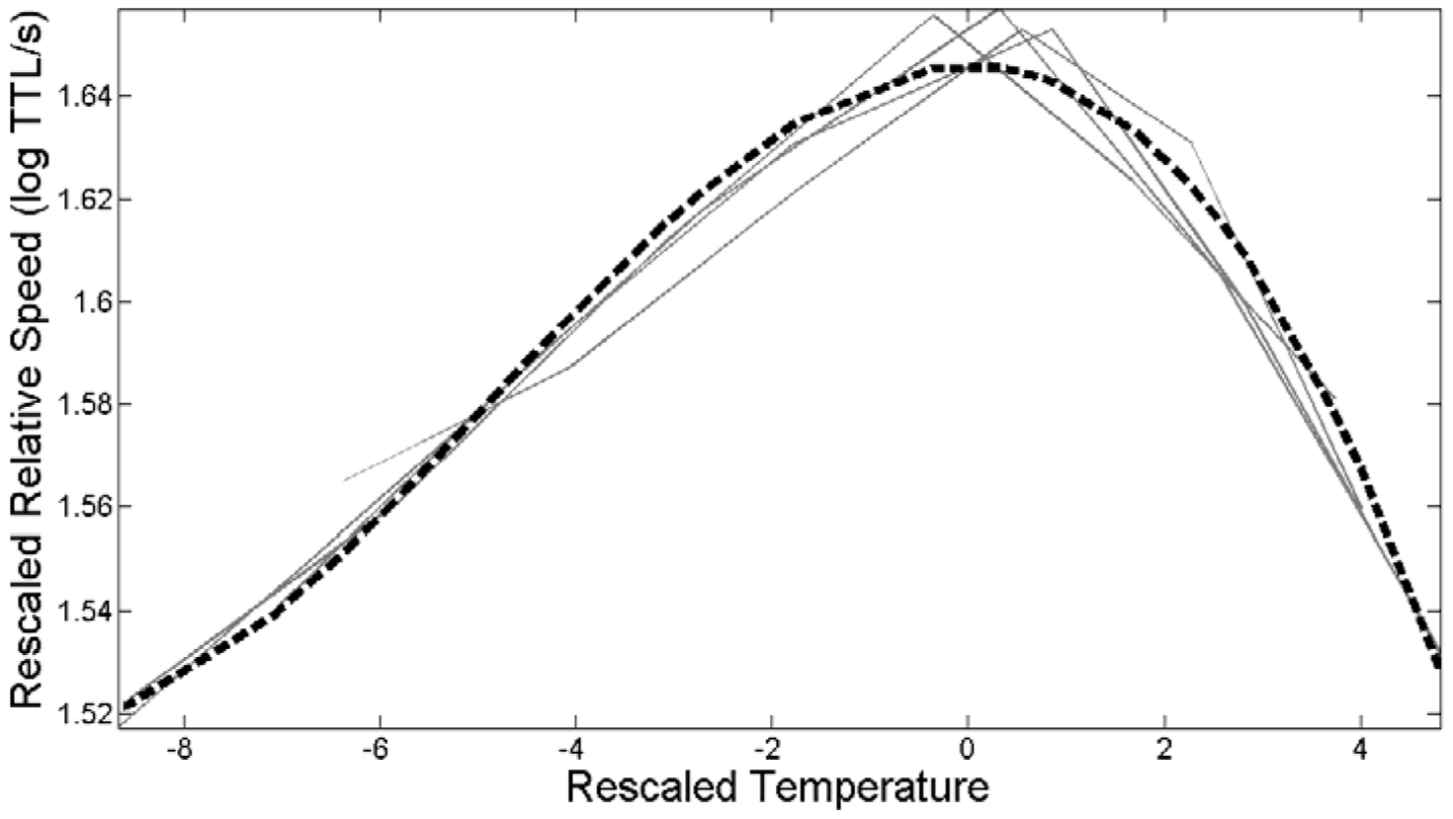
Rescaled thermal performance curves for swimming speed in each treatment with fitted common template shape. Common template shape z(T) is represented by a dashed line nad the treatments by solid lines. Each thermal performance curve of a treatment (i) and temperature were standardized with respect to the estimates of height (h), location (m; T_opt_), and width (w) parameters from the fit to model. Rescaled optimum temperature T_opt_ = 0. (see [46], [51]). Swimming z(T)  = 1.6458–0.004T^2^–0.00023982T^3^+0.000003493T^4^.

**Figure 2:**
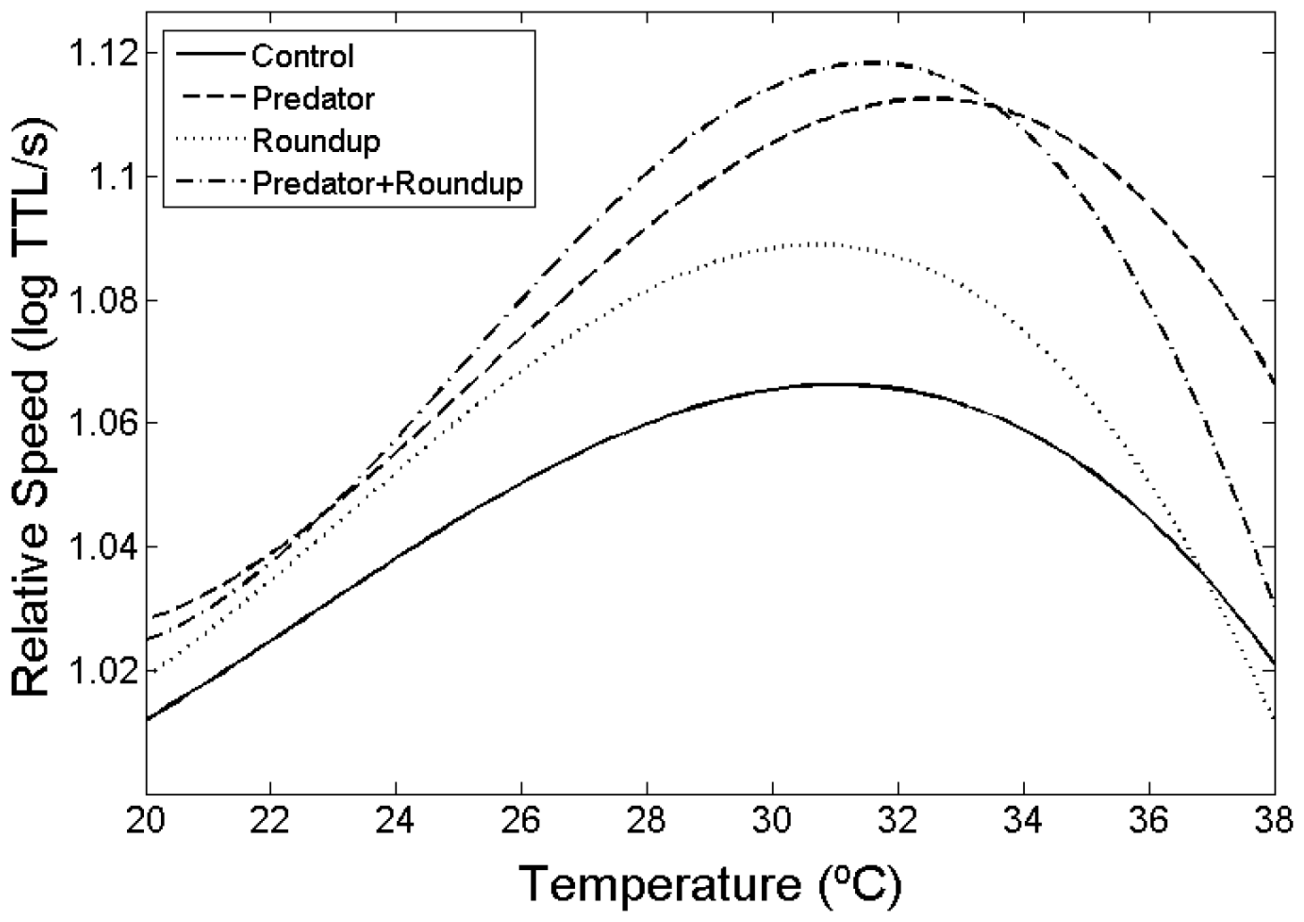
Overall shape of the thermal performance curves for each of the four induction treatments. Each treatment is represented by a thermal performance curve for tadpole swimming speed: control - solid line, predator - dashed line, Roundup - dotted line and predator+Roundup - dash-dot line.

When we test tadpole swimming ability across different water temperatures, we found that swimming burst speed varied with temperature (Table 2). When we used the TMV method on size-corrected performance data, we obtained both a common template curve, which provided a good approximation of the common shape of each treatment's curve (Fig. 1), and a three-parameter shape-invariant model (with the use of a fourth-degree polynomial), which explained over 99% of the variation for swimming speed. Decomposition of the total variation into the three pre-determined directions of variation reveals that TPCs for swimming speed vary mostly in the specialist-generalist (53.27%) direction and the vertical (45.98%) direction, but very little in the horizontal (0.59%) direction. This indicates that tadpoles in the control treatment had a wider swimming TPC than tadpoles exposed to predator cues or the herbicide, even when comparing more traditional measures of curve width (B_95_; Table 4, Fig. 2). Thus, most of the variation in the TPCs is due to specialist-generalist trade-offs and differences in overall performance (faster-slower), rather than changes in T_opt_ (hotter-colder). Indeed, tadpoles raised in the herbicide treatment exhibited only a small decrease in T_opt_ (−0.4°C) while tadpoles raised with predator cues exhibited an increase in T_opt_ (1.5°C). Tadpoles raised with both predators and herbicide exhibited a T_opt_ that was intermediate in magnitude between the latter two treatments but still higher (0.5°C) than tadpoles raised in the control treatment. The only significant difference in T_opt_ was between tadpoles exposed only to herbicide and those exposed only to predator cues (1.8°C; 2-tailed *t*-test, *p*<0.05). Maximal performance (z_max_) was marginally correlated with performance breadth (Pearson's R = −0.95, *p* = 0.051).

**Table 4:** Parameters of thermal performance curves for maximum swimming speed in four treatments, for *Hyla versicolor*, estimated with TMV method (Izem and Kingsolver, 2005) and nlinfit/nlparci functions in Matlab (Mathworks, 2009).

Treatment	TMV parameters						nlinfit/nlparci		
	h^¥^	T_opt_	w	z_max_ 1	z_max_ 2	B_95_	h±SE	T_opt_±SE	w±SE
**Control**	0.12	31.05	1.74	1.07	11.65	18.36	0.12±0.10	31.07±0.76	1.73±0.19
**Predator**	−0.01	32.52	1.47	1.11	12.96	14.29	−0.01±0.09	32.53±0.52	1.47±0.13
**Roundup**	0.00	30.70	1.52	1.09	12.27	14.97	0.00±0.09	30.71±0.65	1.52±0.14
**Predator + Roundup**	−0.11	31.58	1.34	1.12	13.13	12.35	−0.11±0.09	31.58±0.51	1.33±0.11

h^¥^, height (log TTL/s); T_opt_, optimal temperature (°C); w, width (dimensionless); z_max_ 1 (TPC), maximum performance (log TTL/s); z_max_ 2 (TTL/s), maximum performance (TTL/s); B_95_, thermal performance breadth (°C).

Temperature and predator cues both influenced burst speed (Table 5). There was also a significant interaction between predator cues and herbicide. Tadpoles in the control treatment had slower burst speeds accross all temperatures than tadpoles in the other three treatments (all *p*<0.05). Tadpoles raised in the predator treatment were also faster than those from herbicide treatment (*p*<0.05). Furthermore, tadpoles in all treatments containing predator cues or herbicide had higher maximum performance (z_max_) than tadpoles in the control treatment, so that their burst speed at the optimum temperature was higher than the burst speed of tadpoles raised without any cues. These differences in the parameters of the TPCs can be seen as changes in the overall shape of the curves (Fig. 2). Our analysis of burst speed also revealed a significant effect of mesocosms (nested within treatment), however the magnitude of this effect was much smaller than in other effects, such as the interaction of predator cues and herbicide (Table 5). Nevertheless, we checked for burst speed differences among tanks of the same treatment and temperature and we found no significant effect of mesocosm on burst speed, in any of the treatment-temperature combinations (all *p*>0.05).

**Table 5:** ANOVA using burst speed as dependent variable, and temperature, mesocosm, predator cues and Roundup as categorical predictors, with mesocosm nested within the interaction of predator cues and Roundup, for *Hyla versicolor*.

	SS	d.f.	MS	F	p
**Temperature**	0.891	5	0.178	32.17	<0.001
**Predator**	0.106	1	0.106	19.16	<0.001
**Roundup**	0.002	1	0.002	0.38	0.537
Predator*Roundup	0.070	1	0.070	12.65	<0.001
Mesocosm (Predator*Roundup)	0.127	12	0.010	1.92	0.03
Predator*Temperature	0.023	5	0.005	0.83	0.528
Roundup*Temperature	0.017	5	0.003	0.62	0.683
Predator*Roundup*Temperature	0.009	5	0.002	0.33	0.903
**Error**	3.085	546	0.006		

Univariate tests of significance for burst speed. We used Sigma-restricted parameterization and Type III sum of squares.

### Induced morphology of the tadpoles

**Figure 3:**
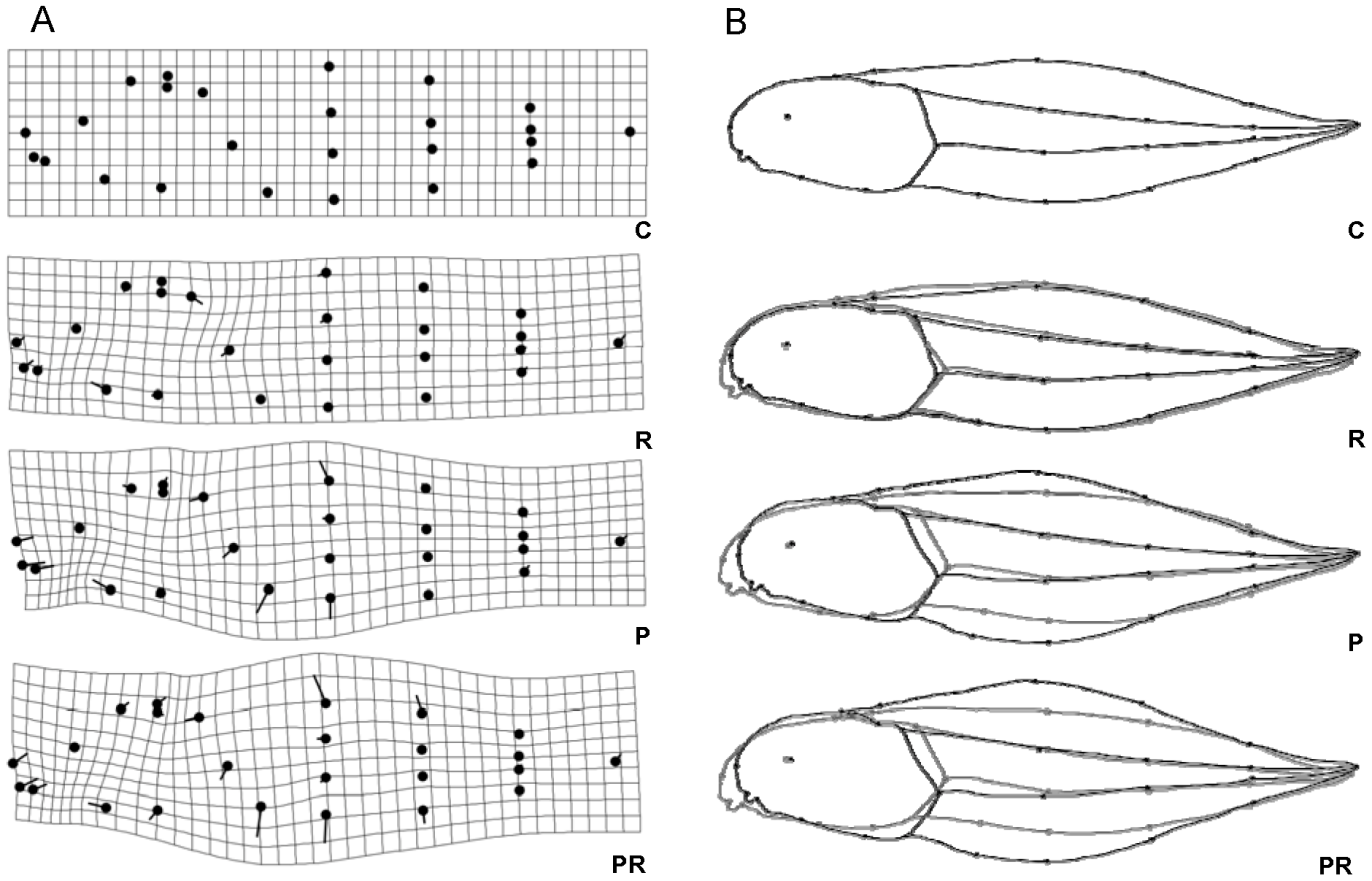
Transformation grids with landmarks and warped outline drawings for each treatment's tadpole shape. a) Transformation grids with landmarks (black dots) and vectors showing direction of variation; b) comparison of warped outline drawings for each treatment shape (black) and control shape (grey). Transformation grids and warped outline drawings were magnified (x5) to better illustrate the differences. C – Control, R – Roundup, P – Predator and PR – Predator + Roundup.

We observed size and shape changes in tadpoles exposed to the herbicide and predator cue treatments (Fig. 3). Predator cues and herbicide had no main effects on tadpole centroid size (Table 6a) but they did have a significant interaction; tadpoles exposed to predator cues + herbicide were smaller than those exposed only to the herbicide or only to the predator cues (both *p*<0.05). Similarly, tadpoles in the control treatment were smaller than those exposed only to the herbicide or only to the predator cues (both *p*<0.05). For geometric morphometric measurements, both predator cues and herbicide influenced tadpole shape (Table 6b) and there was a significant interaction between the two factors. Tadpoles raised in the control treatment differed from those raised in the other three treatments (all *p*<0.05), however these did not differ amongst themselves. For linear measurements, only predator cues significantly influenced shape of tadpoles (Table 6c). Tadpoles raised in predator or predator + herbicide treatment differed from those raised in herbicide or control treatments (all *p*<0.05). Mesocosm effect on either centroid or shape (MI_lin_ or MI_geo_) was non-significant (Table 6). Overall, compared to tadpoles in the control, tadpoles in the other three treatments exhibited relatively shorter bodies. Furthermore, in the two treatments containing predator cues, tadpoles exhibited an increase in their relative tail length and tail depth (Fig. 3). Apart from temperature and predator cues, burst speed was also influenced by tadpole's size, either when using morphometric geometric data (Table 7a) or linear measurements (Table 7b). We also found a significant effect of shape on burst speed when using geometric morphometric data (Table 7a).

**Table 6:** ANOVAs to determine if predator cues and Roundup (including their interaction) influenced size (a; centroid), or shape (b and c) of tadpoles (MI_geo_, for geometric morphometric measurements, or MI_lin,_ for linear measurements, respectively) with mesocosm nested within the interaction of predator cues and Roundup (i.e. mesocosm nested within treatment).

a) Centroid (size)	SS	d.f.	MS	F	p
**Predator**	19.9	1	19.91	0.97	0.326
**Roundup**	4.3	1	4.32	0.21	0.647
Predator*Roundup	521.7	1	521.7	25.38	<0.001
Mesocosm (Predator*Roundup)	423.0	12	35.25	1.72	0.06
**Error**	11386.2	554	20.55		

b) MI_geo_ (shape)	SS	d.f.	MS	F	p
**Predator**	11.77	1	11.766	12.21	<0.001
**Roundup**	5.17	1	5.172	5.37	0.021
Predator*Roundup	5.68	1	5.684	5.90	0.016
Mesocosm (Predator*Roundup)	12.37	12	1.031	1.07	0.383
**Error**	533.95	554	0.964		

c) MI_lin_ (shape)	SS	d.f.	MS	F	p
**Predator**	29.35	1	29.348	30.95	<0.001
**Roundup**	3.32	1	3.317	3.50	0.062
Predator*Roundup	2.46	1	2.463	2.60	0.108
Mesocosm (Predator*Roundup)	8.67	12	0.723	0.76	0.690
**Error**	525.32	554	0.948		

We used Sigma-restricted parameterization and Type III (Effective hypothesis) sum of squares.

**Table 7:** ANCOVA analysis using burst speed as dependent variable, shape variables MI_geo_ (a) or MI_lin_ (b) and tadpole size (centroid) as continuous predictors, alongside temperature, predator cues and Roundup as categorical predictors.

a)	SS	d.f.	MS	F	p
**Predator**	0.068	1	0.068	15.01	<0.001
**Roundup**	0.000	1	0.000	0.01	0.909
**Temperature**	0.197	5	0.039	8.66	<0.001
**Size (Centroid)**	0.167	1	0.167	36.70	<0.001
**Shape (MI_geo_)**	0.129	1	0.129	28.27	<0.001
Predator*Roundup	0.011	1	0.011	2.34	0.127
**Error**	2.544	559	0.005		

b)	SS	d.f.	MS	F	p
**Predator**	0.101	1	0.101	21.24	<0.001
**Roundup**	0.003	1	0.003	0.59	0.443
**Temperature**	0.507	5	0.101	21.30	<0.001
**Size (Centroid)**	0.410	1	0.410	86.09	<0.001
**Shape (MI_lin_)**	0.011	1	0.011	2.37	0.124
Predator*Roundup	0.012	1	0.012	2.45	0.118
**Error**	2.661	559	0.005		

Univariate tests of significance for burst speed. In both models, we used Sigma-restricted parameterization and Type III (Effective hypothesis) sum of squares.

## Discussion

We discovered that predator cues and the herbicide Roundup can affect the thermal physiology of *Hyla versicolor* tadpoles. Predator cues induced tadpoles to have CT_max_ values that were 0.4°C higher whereas the herbicide had no effect. Predator cues and Roundup also influenced the shape of the thermal performance curves, resulting in changes in optimum temperature, performance breadth and maximal performance (Fig. 2). Furthermore, predator cues also induced morphological changes that increased the tadpoles' burst speed.

Roundup, a glyphosate based broad-spectrum systemic herbicide, did not have any effect on CT_max_ estimates of tadpoles. However there have been reports of other contaminants affecting the thermal physiology of vertebrates. Among insecticides, for example, endosulfan (an organochlorine insecticide that affects the central nervous system) and chlorpyrifos (an organophosphate insecticide that inhibits acetylcholinesterase) are known to decrease CT_max_ in fishes [60]. Other environmental contaminants, such as cadmium and copper, can adversely affect the ability of fish to withstand high temperature stress [61], [62]. Whether all of these observations in fishes can be extrapolated to other species of aquatic organisms, such as tadpoles, is yet to be determined. Based on these studies and our own results, it seems that the effects of pesticides on CT_max_ may depend on the type of pesticide, the concentration of the pesticide, and how it affects the organism (i.e. its mode of action). There is the possibility that using higher concentrations of the herbicide might induce a decrease in CT_max_, but higher concentrations will cause tadpole death [63]. Furthermore, the herbicide also did not interfere with the increase in CT_max_ induced by predator cues; tadpoles exposed to predator cues + herbicide had similar CT_max_ values to those exposed only to predator cues.

Different methodological protocols and biological sources can affect estimates of upper thermal tolerances (see [44], [64]). For example, the ramping rate used [65], [66], [67], [68], the selection of end-point [23], variations in previous thermal acclimation [40], ontogenetic stage [43], time of day, and photoperiod [69] all may promote shifts in amphibian upper thermal tolerances. We discovered that predatory cues can also affect CT_max_ estimates of prey. An increase in thermal tolerance of predator-induced tadpoles would cause an increase in their warming tolerance, which is the difference between CT_max_ and maximum temperature of the environment to which an ectotherm is exposed [70], [71]. This means that tadpoles exposed to predator cues would be less susceptible to acute thermal stress than tadpoles that were not exposed to predator cues. In contrast, an exposure to the herbicide, at least at the concentration used in our study, would not affect the warming tolerance of tadpoles.

An exposure to predator cues and the herbicide had interactive effects on tadpole burst speed. The interaction occurred because the herbicide alone and predator cues alone each increased burst speed compared to the control, but the combination of the herbicide and predator cues induced an increase that was not larger than predator cues alone.Therefore, since the combination of the herbicide and predators cues was not additive, in the presence of predator cues, exposure to the herbicide caused no change in burst speed.

The presence of either predator cues or the herbicide narrowed the performance breadth of the TPC while increasing maximal performance. As performance breadth is negatively correlated with maximal performance, we would expect a generalist-specialist trade-off. Tadpoles from a treatment which induced a more specialist curve (as demonstrated by predator cues + herbicide) would perform better at the optimum temperature but gradually decrease in performance, as moving away from the optimum temperature, until reaching a point were tadpoles from a treatment which induced a more generalist curve (as demonstrated by control) would outperform them (see [31], [51]; Table 4). However, we do not see a decline in performance at the extremes of the thermal performance curve, at the tested temperatures, as a result of this trade-off. This observation is confirmed by thermal tolerance data where none of the tadpoles raised in any of the treatments with predator cues or the herbicide had lower CT_max_ than those from the control treatment. Instead, it appears the expected decline in sub-optimal performance resulting from a generalist-specialist trade-off is compensated by the increase in overall performance, so that tadpoles raised in the control treatment always perform, on average, worse than herbicide- or predator-induced tadpoles, at least at the tested temperatures. Therefore, when comparing thermal performance curves, the resulting increase in overall performance was asymmetric, being greater around the optimum temperature and lower at the extreme temperatures.

Surprisingly, predator cues and the herbicide also produced changes in the optimum temperature, but in opposite directions. Of course, the small decrease in optimum temperature caused by the herbicide (0.4°C) may have little or no biological relevance. In contrast, the increase in optimum temperature promoted by predator cues (approximately 1.5°C) may be important, especially when new assessments suggested that environmental impacts will require smaller degrees of global warming than previously thought [72]. Since predator cues increase optimum temperature, the difference between optimum temperature and the environmental temperature should also increase (i.e. thermal safety margins (TSM); see [70]), which would be beneficial to the tadpoles in the current scenario of increasing global temperatures.

Previous studies have demonstrated that changes in the shape or position of thermal performance curves can occur due to acclimation (e.g., [73], [74], [75]) or that thermal performance curves of different locomotor strategies for the same organism can have different shapes (e.g., [46], [76]). In the present study, we demonstrate that the presence of sublethal concentrations of an herbicide and cues from predators can also produce changes in the thermal performance curves and therefore affect how tadpoles respond to environmental temperature changes.

Although it has been documented that predators can affect the behavioral thermoregulation of their prey (e.g., [25]), to our knowledge this is the first study to demonstrate a predator altering the thermal physiology of their prey by increasing CT_max_, increasing the optimum temperature, and producing changes in the thermal performance curves. It has also been demonstrated that Roundup's lethality increases with competition stress [35] and that predator cues can improve tadpole survival when tadpoles are exposed to the herbicide under stratified water conditions [17]. Therefore, one could make the argument that acclimation to predator cues might be beneficial under warmer temperatures. However, we should also keep in mind that predation simultaneously has a negative effect on tadpole populations and can select for particular phenotypes (see [9]). To display a predator-induced phenotype, tadpoles need to detect chemical cues that are released when other tadpoles (particularly conspecifics) are consumed. So, the possible positive effects of predator cues on the thermal physiology, in a global warming scenario, would only be beneficial for those phenotypes that survive predation.

Predator cues in our study induced morphology changes (relative smaller bodies, deeper tails and deeper tail muscle) that were similar to those observed in previous studies (e.g., [77]). These morphological changes likely explain why tadpoles exposed to predator cues swam faster than control tadpoles. Exposure to the herbicide (see figure 3) induced relative smaller bodies, and the observed changes partially resembled the predator-induced phenotype (see also [17]). The induction of relatively deeper tadpole tails by the herbicide was less evident in the current work than in the study of Relyea [17]. However, this may be due to a number of differences in the experimental protocol including the duration of exposure and a substantially different experimental venue.

Predator cues and the herbicide caused interactive effects on tadpole size. Tadpoles exposed to predator cues + herbicide were smaller than those exposed only to the herbicide or only to predator cues. Tadpoles raised in the control treatment also tended to be smaller than those exposed only to the herbicide or only to predator cues. This may explain why tadpoles from the herbicide treatment also swam faster than tadpoles from the control treatment. As a result, all three treatments had better overall swimming performance than in control, with increase in burst speed related to the magnitude of morphology change (more induction, higher performance) and size. Furthermore, predator-induced morphology changes can be reversed if cues are removed [41]. As a result, some of the changes in the thermal performance curve may also be reversible. If so, in the absence of cues, the predator- and herbicide-induced TPC shapes would revert back to the original curve (i.e. the control curve).

The mechanism underlying the ability of the herbicide to induce morphological changes in tadpoles is still unknown. It has been suggested that the herbicide may be interfering with the stress hormones that induce anti-predator defenses [78] or that herbicides and predator cues activate shared endocrinological pathways [17]. We have demonstrated that predator cues and the herbicide can affect the thermal physiology of tadpoles, although not all changes occur in the same direction. However, the mechanisms behind these thermal physiology changes are also unknown, with possible scenarios arising from our results: a) herbicide interferes only with the stress hormones that induce anti-predator defenses; b) they do not share the same physiological pathways, or at least not all of them; c) they both activate shared endocrinological pathways but predator cues also indirectly activate temperature-stress response mechanisms; or d) stress response mechanisms are more general than previous thought and predator-induced stress produces similar physiological responses as temperature-induced stress.

## Conclusions

Apart from inducing morphology changes, predator cues promoted an increase in CT_max_ and optimum temperature of *Hyla versicolor* tadpoles. As such, in the presence of predators, we can expect tadpoles to have greater warming tolerance and broader thermal safety margins. These changes might indirectly help tadpoles cope with increasing environmental temperatures. The herbicide Roundup is not only toxic to amphibians (and lethal over certain concentrations), but it also produces changes in morphology [17]. With this work, we now know that it also interferes, to some extent, with the thermal physiology of tadpoles (in particular in the thermal performance curves), although the effect on warming tolerance and thermal safety margins appears to be marginal. However, Roundup is just one of hundreds of chemicals currently used in anthropogenic activities (e.g., agriculture) and tadpoles can face predation by a wide variety of predator species. Because combinations of pesticides, which are a common situation in natural environments, can have greater impacts than each pesticide alone [79], future studies should test whether combinations of pesticides and predators could have different effects on the thermal physiology of organisms.

In the current scenario of climate change, it is important that we understand the physiological mechanisms underlying tolerance to abiotic stress [80], [81] and the sensitivity of organisms to changes in the environment [80], [82]. However, it also is important that we understand the indirect effects of physiological responses (in particular thermal physiology) on species interactions, such as predation, competition and disease transmission [2]. Therefore, understanding the plasticity of thermal performance curves and thermal limits (CT_max_ and CT_min_) and how these parameters are altered by environmental stressors may be critical to understanding how physiological variation can influence a species' response to climate change [83].

## Supporting Information

Methods S1 **Appendices 1–4.** Appendix 1, Detailed information on laboratory conditions for rearing tadpoles during acclimation for the experiments. Appendix 2, Description of method and apparatus used for measuring CT_max_. Appendix 3, TMV method equation for calculating thermal performance curve's parameters. Appendix 4, Description of the side-view landmarks and semi-landmarks, and linear measurements in a hypothetical tadpole.
(DOC)

Raw Data S1 **CT_max_ and swimming performance database.**
(XLS)

TPS Data S1 **Geometric morphometry database.**
(TPS)
