# Supplementary material for: Swimming with Predators and Pesticides: How Environmental Stressors Affect the Thermal Physiology of Tadpoles
Source: PLoS One. 2014 May 28;9(5):e98265. doi: 10.1371/journal.pone.0098265 (PMC4037208; doi:10.1371/journal.pone.0098265)
Supplement: Methods S1 — Appendices 1–4. Appendix 1, Detailed information on laboratory conditions for rearing tadpoles during acclimation for the experiments. Appendix 2, Description of method and apparatus used for measuring CTmax. Appendix 3, TMV method equation for calculating thermal performance curve's parameters. Appendix 4, Description of the side-view landmarks and semi-landmarks, and linear measurements in a hypothetical tadpole. (DOC) [file pone.0098265.s001.doc]

**Appendix 1.** Detailed information on laboratory conditions for rearing tadpoles during acclimation for the experiments.

On day 10, we brought sets of tadpoles into the laboratory to allow them to acclimate to lab conditions for 4 to 5 d before testing them for CT_max_ and T_opt_. This acclimation period was chosen since previous research in adult amphibians revealed that 2 to 3 d are required to stabilize CT_max_ after a large change in acclimation temperature as occurs with outdoor environments (Hutchison, 1961; Brattstrom, 1968). While acclimating for the laboratory experiments, we needed to maintain each treatment’s cues to avoid the loss of the induction (Relyea 2003). Therefore, tadpoles brought into the lab were kept in smaller plastic containers (40 x 25 x 20 cm) with approximately 10 L of water and a small subset of the leaf litter from the original pool from each set of tadpoles. We also added predator cages to all of the lab containers but only those tadpoles assigned to predator treatments had cages that contained a dragonfly nymph. These nymphs were fed ~100 mg grey treefrog tadpole biomass, every 2 d. Note that although the amount of grey treefrog biomass was smaller, the water volume of the lab containers was also much smaller thus maintaining the saturation of the plasticity response. Each container had ~15 tadpoles which were fed rabbit pellets *ad libitum*. The lab was held at a temperature of 20 °C with a 12L:12D photoperiod.

**Appendix 2.** Description of method and apparatus used for measuring CT_max_.

The testing apparatus consisted of a pool fit to a water bath with several 250-ml test containers filled with 200 ml of dechlorinated water at the acclimation temperature of 20 °C. The pool was heated at the determined rate using heating resistances attached to a temperature controller and temperature was homogenized using water pumps to create water movement in the pool. After achieving CT_max_, we transferred tadpoles to cooler water (~20 °C) to allow recovery, after which they were weighed. We only tested tadpoles between 31-37 Gosner stages (Gosner, 1960) since tadpoles over 38 Gosner stage have reduced thermal tolerances (Sherman, 1980). Each individual was tested only once and each 250-ml container had only one tadpole per trial.

**Appendix 3.** TMV method equation for calculating thermal performance curve’s parameters.

The TMV method employs a polynomial function to decompose variation among TPCs into three predetermined modes of variation with biological connotation: vertical shift (faster-slower), horizontal shift (hotter-colder), and specialist-generalist trade-offs (Huey and Kingsolver, 1989; Izem and Kingsolver, 2005). Using a shape-invariant model (Eq. 1), we obtained one parameter for each direction of variation, i.e., height, optimal temperature, and width of TPC (Izem and Kingsolver, 2005):

$z_{i}\left(T\right)=\frac{1}{w_{i}}z\left[\frac{1}{w_{i}}\left(T-T_{opt,i}\right)\right]+h_{i}$ Eq. (1)

In Eq. (1), z_i_(T) is performance at temperature T for treatment i, z represents the common template shape of the curves, h_i_ is the height of TPC, T_opt,i_ is the optimal temperature, and w_i_ is the width of TPC (also represents the specialist-generalist trade-off and is dimensionless, see Izem and Kingsolver 2005).

**Appendix 4.** Description of the side-view landmarks and semi-landmarks, and linear measurements in a hypothetical tadpole.


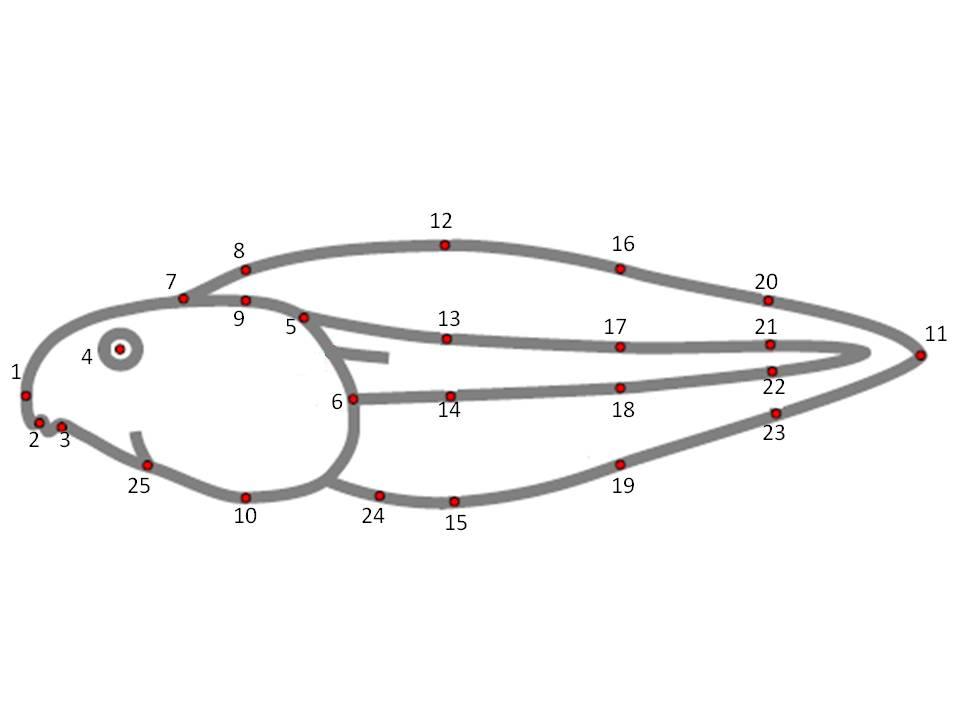


#1 most anterior point of snout; #2 center of partially-opened mouth; #3 junction of posterior edge of lower labium and body wall; #4 center of pupil; #5 point where upper edge of tail muscle meets body; #6 point where bottom edge of tail muscle meets body; #7 point at which dorsal tail fin attaches to top of body; #8 highest point of body or tail fin 2/3rds between #1 and #6; #9 dorsal edge of body 2/3rds between #1 and #6; #10 ventral edge of body 2/3rds between #1 and #6; #11 tip of tail fin; #12 upper edge of tail fin 1/4 between #6 and #11; #13 top of tail muscle 1/4 between #6 and #11; #14 bottom of tail muscle 1/4 between #6 and #11; #15 ventral edge of tail fin 1/4 between #6 and #11; #16 upper edge of tail fin halfway between #6 and #11; #17 top of tail muscle halfway between #6 and #11; #18 bottom of tail muscle halfway between #6 and #11; #19 ventral edge of tail fin halfway between #6 and #11; #20 upper edge of tail fin 3/4 between #6 and #11; #21 top of tail muscle 3/4 between #6 and #11; #22 bottom of tail muscle 3/4 between #6 and #11; #23 ventral edge of tail fin 3/4 between #6 and #11; #24 point where center of anus meets lower edge of tail fin; #25 lower edge of body at anterior gut margin. Semilandmarks (#12 to #23) were defined by drawing a line between point #6 and #11 and perpendicular lines 25%, 50% and 75% of the distance along this line. Then, each landmark was placed at the intersections of these lines with the ventral and dorsal margins of the tail fin and tail muscle. Semilandmarks #8, #9 and #10 were generated similarly by drawing a perpendicular line 2/3 between point #1 and #6, and the semilandmarks were placed in the intersection of the line with tail fin and dorsal/ventral edges of head/body.

Linear measurements: total tadpole length (TTL, distance between #1 and #11), body length (BL, distance between #1 and #6), body depth (BD, deepest point of the body), tail length (TL, distance between #6 and #11), muscle depth (MD, deepest point of the muscle) and tail depth (TD, maximum depth of the tail fin).
